# *ntRoot*: Genetic Ancestry Inference Across Diverse Genomic Data Types

**DOI:** 10.1002/cpz1.70491

**Published:** 2026-10-29

**Authors:** Lauren Coombe, Taghrid Aloraini, Inanc Birol, René L. Warren

**Affiliations:** ^1^ Department of Basic and Translational Research British Columbia Cancer Research Institute Vancouver Canada; ^2^ Department of Medical Genetics University of British Columbia Vancouver Canada

**Keywords:** genetic ancestry, genome assembly, SNP array, whole exome sequencing, whole genome shotgun sequencing

## Abstract

Genetic ancestry is encoded in our genomes, reflecting the genealogical history of individuals and populations. This information is increasingly recognized as an important variable in large‐scale population studies, where it is used to correct for population stratification, as well as in clinical applications, since disease risk can vary across populations. However, many studies either fail to account for ancestry, or rely on self‐reported ancestry, which is often incomplete or inconsistent. Most cohort‐level studies utilize single‐nucleotide polymorphism (SNP) arrays, whole exome sequencing (WES), or whole genome sequencing (WGS), all of which capture genetic variation informative of ancestry. Therefore, there is great value in inferring ancestry directly from these heterogeneous data types. Previously, we developed *ntRoot*, a scalable tool for inferring global and local ancestry from WGS reads or assembled genomes. In newer releases, we expanded the utility of *ntRoot* to also infer ancestry from WES and SNP array data, making the tool flexible and robust to the wide diversity of sequencing‐ and array‐based data used in large‐scale genetics studies. The streamlined execution of *ntRoot* enables fast (<70 min for the *ntRoot* step of each protocol) and scalable ancestry inference across a wide variety of research workflows. © 2026 The Author(s). *Current Protocols* published by Wiley Periodicals LLC.

**Basic Protocol 1**: Running *ntRoot* with WGS data

**Basic Protocol 2**: Running *ntRoot* with assembled genomes

**Basic Protocol 3**: Running *ntRoot* with WES data

**Basic Protocol 4**: Running *ntRoot* with Illumina SNP array data

**Basic Protocol 5**: Running *ntRoot* with variant call format files from variant calling pipelines

**Support Protocol**: Installing dependencies for the Basic Protocols

## INTRODUCTION

Our genomes reflect our genetic ancestry, revealing insights into our genealogical history. A wealth of ancestry information can be gained by analyzing human DNA, including deeper insights into population‐specific disease risk and population structure, and unraveling confounding factors in genome‐wide association studies (GWAS) (Bird et al., 2025; Padhukasahasram, 2014). These insights can have direct impacts in personalized medicine to better understand disease risk and progression for patients (Smart et al., 2017). Despite the importance of accurately and comprehensively characterizing genetic ancestry, many studies fail to properly account for it due to incomplete or missing information.

This challenge is exacerbated by the diversity gap in human genomics research. Over 80% of participants in GWAS are of European ancestry, while African, Latin American, and other underrepresented populations are only marginally included (Corpas et al., 2025; Pomales‐Matos et al., 2025). This Eurocentric bias limits the portability of polygenic risk scores and reduces the diagnostic accuracy of variant interpretation for non‐European patients (Ojewunmi & Fatumo, 2025). Closing this gap depends not only on improved recruitment, but also on the ability to infer accurate ancestry labels to existing and incoming data (Kaseniit et al., 2020). Addressing this need requires methods capable of operating on the rapidly expanding volumes of sequencing data generated by population‐scale studies, supporting the heterogeneous data types used in large‐scale genomic studies, and resolving ancestry at the continental (super‐population) level used by contemporary reference resources, including the 1000 Genomes Project (1000 Genomes Project Consortium et al., 2015) and gnomAD (Chen et al., 2024).

Genetic ancestry inference can be divided into two general categories: global ancestry inference (GAI) and local ancestry inference (LAI) (Akgun & Rajabli, 2026). GAI summarizes ancestry proportions across the entire genome, whereas LAI provides more fine‐grained ancestry labels across smaller stretches of the input chromosomes. In our multicultural world, many genomes are admixed to varying degrees, which is reflected in the presence of different reference population ancestries in different regions of their genomes (Lerga‐Jaso et al., 2025). Most bioinformatics tools for inferring ancestry are classified as analyzing GAI, e.g., ADMIXTURE (Alexander et al., 2009), STRUCTURE (Pritchard et al., 2000), or SNVstory (Bollas et al., 2024), or LAI, e.g., RFMix (Maples et al., 2013), Tractor (Atkinson et al., 2021), or FLARE (Browning et al., 2023), but there is great value in a single tool that can analyze ancestry at both resolutions. In addition to estimating ancestry, LAI has also become a step in a wide range of downstream analyses, including admixture mapping, ancestry‐aware GWAS, ancestry‐specific expression quantitative trait locus (eQTL) discovery, and polygenic score construction for admixed individuals (Sun et al., 2025). Benchmarks show that LAI's accuracy for admixed populations depends on reference panel composition, phasing method, and ancestry‐specific representation. Interestingly, phase‐free methods have shown improved robustness compared to phase‐based approaches, particularly by reducing errors related to phasing, a concern that can significantly affect analyses of recently admixed populations (Avadhanam & Williams, 2025). Inferring underrepresented ancestral components, such as Amerindigenous ancestry, remains particularly challenging (Honorato‐Mauer et al., 2025).

Studies of human populations can generate a range of different genomic data, from whole genome sequencing (WGS) or whole exome sequencing (WES) to single‐nucleotide polymorphism (SNP) arrays. All these data types capture ancestry information to varying degrees, but not all ancestry prediction tools can leverage the diverse data across this spectrum. Many tools require variant files as input to their pipelines, but generating these files from the raw data is not always trivial. Therefore, a streamlined, user‐friendly, and scalable tool capable of handling multiple data types directly while supporting both GAI and LAI output would be a valuable contribution to the community.

In this article, we describe running *ntRoot* (Warren et al., 2025), a scalable tool for ancestry inference which provides global ancestry inference as well as local ancestry predictions per genome tile. Its utility at population scale has recently been demonstrated through its application to WGS data from 10,250 participants in the Canadian HostSeq cohort (Warren et al., 2026). We will demonstrate how *ntRoot* can be used with multiple different input data types, including WGS data, genome assemblies, WES data, SNP arrays, and variant call format (VCF) files from a standard variant calling pipeline. We believe that *ntRoot's* flexibility in inferring ancestry at different resolutions and with different data types will enable genetic ancestry predictions for a wide variety of studies.

## STRATEGIC PLANNING

### Hardware

*ntRoot* can be run from the command‐line on macOS or Linux operating systems with sufficient random‐access memory (RAM) (up to 46 GB in our protocols, see Table 1). The amount of RAM required for running *ntRoot* depends on the input data provided, as well as the reference genome size and sequencing coverage (where applicable). In Table 1, we show examples of wall‐clock time and peak RAM usage for different input data types. The run times will also depend on the number of threads used by *ntRoot*, which can be adjusted using the “‐t” parameter in the *ntRoot* command.

**Table 1:** Example Run Time, Peak RAM and Disk Space Usages When Running the ntRoot Step of the Protocols*^a^*

Basic Protocol	Input data type	Input data specifications	Run time (min)*^b^* *^,^* *^c^*	Peak RAM (GB)	Disk space (GB)*^d^*
1	Whole genome sequencing reads	∼30‐fold, 2 × 150 bp reads; *n* = 758.2 × 10^6^ reads	70	45.6	1.4
2	Genome assembly	Pseudo‐haploid human genome (3 Gbp)	30	14.2	1.2
3	Whole exome sequencing reads	∼110‐fold, 2 × 90 bp reads; *n* = 56.8 × 10^6^ reads	10	9.4	0.04
4	SNP array	649,046 markers	7	0.09	0.1
5	3^rd^ party VCF file	5,066,913 variants; 160 MB compressed file	10	0.09	1.4

a All input data is from human individuals.

b Using 24 threads.

c Listed run times are only for the *ntRoot* step, and do not include the time for tool installation, data download, or any data pre‐processing.

d Only includes disk space used by final output files generated by the *ntRoot* command.

### Software

*ntRoot* can be installed using the conda package manager. Alternatively, *ntRoot* can be run using a Docker image. For more information about both options, please see the Support Protocol below.

*NOTE*: Appropriate informed consent is necessary for obtaining and use of human study material.

## Basic Protocol 1 RUNNING *ntRoot* WITH WHOLE GENOME SEQUENCING DATA

Basic Protocol 1 describes running *ntRoot* using WGS reads as input. First, *ntRoot* runs ntCard (Mohamadi et al., 2017), which estimates the *k*‐mer (substring of length *k*) frequency histogram from the input sequencing reads. ntStat (Kazemi et al., 2026) uses the ntCard‐derived histogram, along with the sequencing reads, to infer parameters such as the minimum *k*‐mer abundance threshold, and loads the input sequencing reads into a probabilistic data structure called a Bloom filter. This Bloom filter is then input to ntEdit (Warren et al., 2019) along with the human reference genome sequence and is used to detect single nucleotide variants (SNVs) between the input sequencing reads and the reference genome sequence. Finally, *ntRoot* cross‐references these detected variants with an integrated variant call set (IVC) file built from the 1000 Genomes Project (1000 Genomes Project Consortium et al., 2015) to infer the super‐population ancestry of the input individual.

### Necessary Resources

#### Hardware

64‐bit Linux or macOS operating system with sufficient RAM and diskspace (see Table 1)

##### Software

The listed software should be installed:

ntRoot (v1.2.4+): https://github.com/BirolLab/ntRoot
ntEdit (v2.1.1+): https://github.com/BirolLab/ntedit
snakemake: https://snakemake.readthedocs.io/en/stable/
samtools: https://www.htslib.org/
wget: https://www.gnu.org/software/wget/
*For step‐by‐step instructions describing how to install these dependencies, please see the Support Protocol. Ensure that all dependencies are available in your PATH environment variable*.

##### Files

Short paired‐end whole genome sequencing reads (in FASTQ format)

Human reference genome (in FASTA format)

IVC file from the 1000 Genomes Project (in VCF format)

*All the files can be uncompressed or compressed (e.g., with the gzip, pigz or bgzip algorithms). To illustrate the VCF file format, a screenshot is provided in* *Figure* *1*.

**Figure 1:**
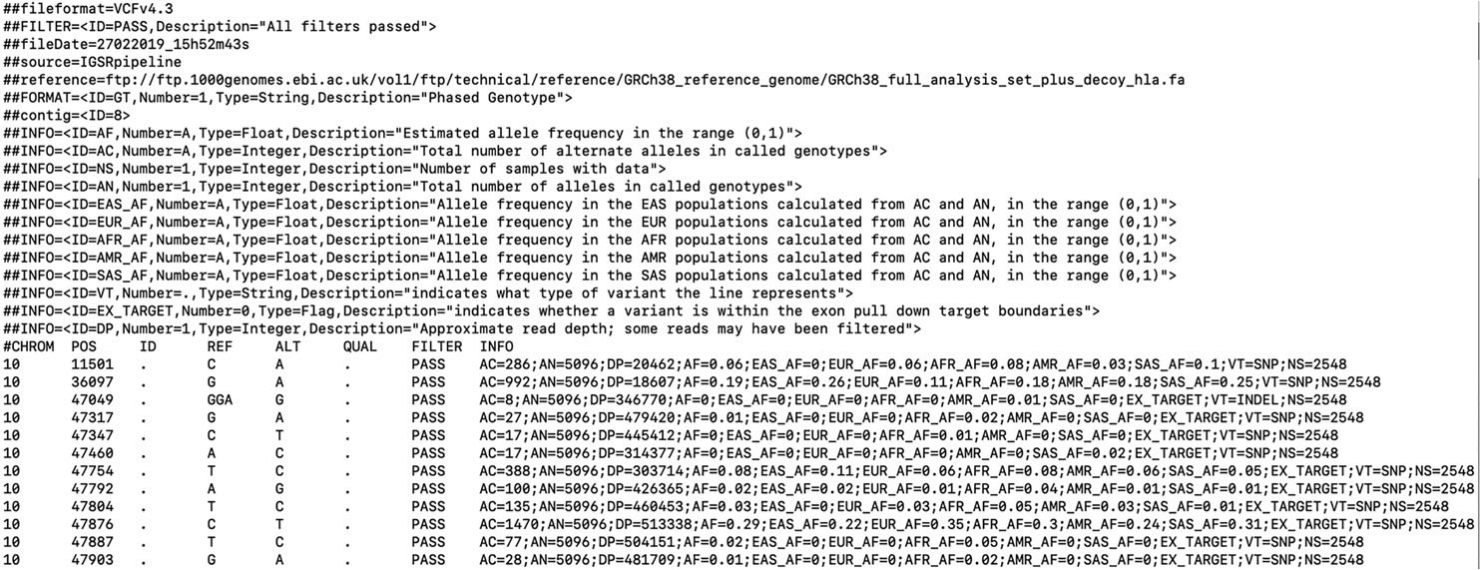
Screenshot of the format of the integrated call set variant file.

1 Prepare the working directory.


mkdir ntroot1 && cd ntroot1

2 Download the sample whole genome sequencing reads.


nohup /usr/bin/time -p wget ftp://ftp.sra.ebi.ac.uk/vol1/fastq/ERR324/002/ERR3242132/ERR3242132_1.fastq.gz &> reads1.log &
nohup /usr/bin/time -p wget ftp://ftp.sra.ebi.ac.uk/vol1/fastq/ERR324/002/ERR3242132/ERR3242132_2.fastq.gz &> reads2.log &

  The ∼30‐fold coverage sequencing reads will be downloaded to your current working directory. Using “nohup” in the above commands allows the files to be downloaded simultaneously. The downloads will each take ∼30 min.
  The downloaded reads are paired‐end Illumina sequencing reads provided by the 1000 Genomes Project for sample HG00864, which has self‐reported East Asian ancestry (Zheng‐Bradley et al., 2017). Ensure that both files have finished downloading before moving onto the next step.
3 Download the human reference genome and IVC files.


nohup /usr/bin/time -p wget https://zenodo.org/records/22151063/files/ntroot_supplementary_zenodo.tar.gz &> download-supplementary.log &

  Ensure that the tar.gz file has finished downloading before proceeding to extracting the files in the next step.
4 Extract the human reference genome and IVC files.


tar xvzf ntroot_supplementary_zenodo.tar.gz

5 Run *ntRoot* using the downloaded whole genome sequencing reads.


ntroot -r ntroot_supplementary_zenodo/data/GRCh38.fa.gz --reads ERR3242132 --lai -t 24 -k 55 -l ntroot_supplementary_zenodo/data/1000GP_integrated_snv_v2a_27022019.GRCh38.phased_gt1.vcf.gz

  The ntRoot command will take ∼1 hr to complete when using 24 threads. Depending on the number of threads available on your machine, you can adjust the ‐t parameter higher or lower.
6 Check the generated output log file to ensure that *ntRoot* completed successfully.
  A successful run will have the message “Done ntRoot!” at the end of the log file.
7 View the global genetic ancestry inference file produced by *ntRoot*.
  The expected global genetic ancestry predictions will be output in a file named ERR3242132_ntedit_k55_variants.vcf_ancestry-predictions_tile5000000.tsv. The super‐populations from the input IVC file are shown in the first column: EAS (East Asian), AFR (African), AMR (Admixed American), SAS (South Asian), and EUR (European). To compute the LAI fraction, each genome tile (default size --tile 5000000 bp) is assigned to a super‐population using the computed variants compared to the IVC file. The fraction is then calculated for each super‐population as the length of tiles assigned to that label divided by the total genome length. Detailed definitions of the statistics in the global ancestry output file are listed in Table 2. The expected contents of this file are shown in Table 3. In this example, the predicted global super‐population label is East Asian. Small deviations in the numbers are expected across different runs.

**Table 2:** Definitions of the Statistics in the Global Ancestry Inference Output File Generated by ntRoot

Statistic	Definition
LAI fraction (tile:5000000 bp)	The reference is split into tiles (default size --tile 5000000 bp), then each genome tile is assigned to a super‐population using the computed variants compared to the IVC file; the LAI fraction is then calculated for each super‐population as the length of tiles assigned to that label divided by the total genome length, expressed as a percentage; if a given tile has ambiguous ancestry, it will be assigned to the category “Unknown” instead of a super‐population
GAI score	$GAIscore=\bar{AF}\times R_{nz}$, where $\bar{AF}$ is the average allele frequency across all SNVs for the given label, and *R*_*nz*_ is the non‐zero SNV rate; *R*_*nz*_ is defined as $R_{nz}=\frac{nz\;AF\;SNV\;count}{total\;nz\;AF\;SNV\;count}$, or the total number of SNVs (single nucleotide variants) with non‐zero allele frequency for the given label, divided by the total number of SNVs with non‐zero allele frequency
Total SNV count	The total number of SNVs (single nucleotide variants) detected by the ntEdit SNV‐calling step that overlap with the IVC file
Non‐zero AF SNV count	The total number of SNVs detected by the ntEdit SNV‐calling step that overlap with the IVC file and have a non‐zero allele frequency for the given super‐population label

**Table 3:** Expected Results From Running Basic Protocol 1, *^a^*

GAI super‐population	LAI fraction (tile:5000000 bp)	GAI score	Total SNV count	Non‐zero AF SNV count
EAS	94.40%	0.4925	2,863,566	2,738,589
AFR	2.98%	0.4126	2,863,566	2,704,785
AMR	1.23%	0.4423	2,863,566	2,711,658
SAS	1.23%	0.4456	2,863,566	2,702,532
EUR	0.18%	0.4202	2,863,566	2,652,128

a Super‐population label abbreviations: AMR, Admixed American; AFR, African; EUR, European; SAS, South Asian; EAS, East Asian.

8 View the local ancestry inference file generated by *ntRoot*.
  Detailed local ancestry inference (LAI) information will be generated in the file ERR3242132_ntedit_k55_variants.vcf_ancestry-predictions-tile-resolution_tile5000000.tsv. This file tallies the ancestry predictions and ntRoot score per reference genome tile (default --tile 5000000 bp). The first 10 tiles from chromosome 1 are shown in Table 4. Some minor deviations from these numbers are expected across different runs. The chrom, start and end columns refer to the chromosome and start/end coordinates of the genomic tile in the reference genome sequence. There is a LAI score per super‐population, which is calculated similarly to the GAI score described in Table 2, but the score is computed per tile. The ancestry_prediction column denotes the predicted super‐population for that tile based on the computed scores.

**Table 4:** The First 10 Chromosome 1 Tiles in the LAI Inference File "ERR3242132_ntedit_k55_variants.vcf_ancestry-predictions-tile-resolution_tile5000000.tsv" are Shown*^a^*

chrom	start	end	ancestry_prediction	AFR‐score	AMR‐score	EAS‐score	EUR‐score	SAS‐score
1	1	5000000	EAS	0.0162	0.0172	0.0192	0.0159	0.0175
1	5000001	10000000	EAS	0.0176	0.0174	0.0200	0.0165	0.0170
1	10000001	15000000	EAS	0.0141	0.0143	0.0162	0.0137	0.0147
1	15000001	20000000	EAS	0.0161	0.0175	0.0197	0.0169	0.0171
1	20000001	25000000	EAS	0.0160	0.0185	0.0206	0.0181	0.0185
1	25000001	30000000	EAS	0.0155	0.0167	0.0186	0.0155	0.0167
1	30000001	35000000	EAS	0.0151	0.0159	0.0181	0.0149	0.0167
1	35000001	40000000	EAS	0.0141	0.0152	0.0173	0.0133	0.0147
1	40000001	45000000	EAS	0.0150	0.0157	0.0182	0.0154	0.0163
1	45000001	50000000	EAS	0.0159	0.0170	0.0193	0.0162	0.0174

a Super‐population label abbreviations: AMR, Admixed American; AFR, African; EUR, European; SAS, South Asian; EAS, East Asian.

## Basic Protocol 2 RUNNING *ntRoot* WITH ASSEMBLED GENOMES

Basic Protocol 2 describes running *ntRoot* using the complete or draft genome sequence assembly from an individual (Warren et al., 2025). In this mode, *ntRoot* will first run ntCard to estimate the number of distinct *k*‐mers (substrings of length *k*) in the input genome assembly. Then, *ntRoot* will load the *k*‐mers from the input genome assembly into a probabilistic data structure called a Bloom filter, using the ntCard *k*‐mer estimation to guide the size of this data structure. Then, this Bloom filter will be supplied to ntEdit along with the reference genome sequence and the IVC file and will be used to infer SNVs between the assembled genome sequence and the reference genome. Then, *ntRoot* proceeds with the same steps as in Basic Protocol 1: the detected variants are used to infer global and local genetic ancestry.

### Necessary Resources

#### Hardware

64‐bit Linux or macOS operating system with sufficient RAM and diskspace (see Table 1)

##### Software

The listed software should be installed:

ntRoot (v1.2.4+): https://github.com/BirolLab/ntRoot
ntEdit (v2.1.1+): https://github.com/BirolLab/ntedit
snakemake: https://snakemake.readthedocs.io/en/stable/
samtools: https://www.htslib.org/
wget: https://www.gnu.org/software/wget/
*For step‐by‐step instructions describing how to install these dependencies, please see the Support Protocol. Ensure that all dependencies are available in your PATH environment variable*.

##### Files

Genome assembly file (in FASTA format)

Human reference genome (in FASTA format)

IVC file from the 1000 Genomes Project (in VCF format)

*All files can be uncompressed or gzipped. The expected format for the IVC file is shown in* *Figure* *1*.

1 Prepare the working directory.


mkdir ntroot2 && cd ntroot2

2 Download the example genome assembly file.


wget https://ftp.ncbi.nlm.nih.gov/genomes/all/GCA/000/002/125/GCA_000002125.1_HuRef/GCA_000002125.1_HuRef_genomic.fna.gz

  To illustrate running ntRoot with an input genome assembly file, we use the publicly available HuRef whole genome assembly. The HuRef assembly is the genome of Dr. Craig Venter, who has European ancestry (Levy et al., 2007).
3 Download the human reference genome and IVC file.
  a. If you have already completed Basic Protocol 1, use the human reference genome and IVC files downloaded and extracted in Basic Protocol 1, steps 3 and 4. Make a soft link to this downloaded folder in the current working directory.

ln -s ../ntroot1/ntroot_supplementary_zenodo

  If the directory ntroot_supplementary_zenodo is in a different directory to the one listed in the example command, adjust the path accordingly.
   
  b. If you do not already have the human reference genome and IVC files downloaded, follow Basic Protocol 1, steps 3 and 4.
4 Run *ntRoot*.


ntroot -r ntroot_supplementary_zenodo/data/GRCh38.fa.gz --genome GCA_000002125.1_HuRef_genomic.fna.gz -l ntroot_supplementary_zenodo/data/1000GP_integrated_snv_v2a_27022019.GRCh38.phased_gt1.vcf.gz -k 55 -t 24 --lai

  Depending on the specifications of your machine, you can adjust the number of threads used by ntRoot (‐t). When using 24 threads, the ntRoot command will take ∼30 min to complete, depending on the specifications of your computer system.
5 Check the *ntRoot* output log file to ensure that the process completed successfully.
  If the ntRoot run was successful, you should see the message “Done ntRoot!” at the end of your log file.
6 View the global ancestry inference file generated by *ntRoot*.
  The global ancestry inference data generated by ntRoot will be in the file GCA_000002125.1_HuRef_genomic.fna.gz_ntedit_k55_variants.vcf_ancestry-predictions_tile5000000.tsv. The expected results are shown in Table 5, and descriptions of the columns can be found in Table 2. In this example, the predicted global ancestry super‐population label is European.

**Table 5:** Results of Running ntRoot Using the Example HuRef Genome Assembly in Basic Protocol 2, *^a^*

GAI super‐population	LAI fraction (tile:5000000 bp)	GAI score	Total SNV count	Non‐zero AF SNV count
EUR	53.24%	0.4899	2,101,385	1,989,644
AMR	21.72%	0.4853	2,101,385	2,008,231
AFR	12.78%	0.4469	2,101,385	2,005,698
SAS	11.21%	0.4782	2,101,385	1,981,015
EAS	1.05%	0.4494	2,101,385	1,882,887

a Super‐population label abbreviations: AMR, Admixed American; AFR, African; EUR, European; SAS, South Asian; EAS, East Asian.

7 View the local ancestry inference file generated by *ntRoot*.
  The local ancestry inference generated by ntRoot will be in the file GCA_000002125.1_HuRef_genomic.fna.gz_ntedit_k55_variants.vcf_ancestry-predictions-tile-resolution_tile5000000.tsv. The first 10 tiles for chromosome 1 are shown in Table 6. Detailed descriptions of the columns of this file can be found in Basic Protocol 1, step 8.

**Table 6:** First 10 Chromosome 1 Tiles in the Local Ancestry Inference File Generated by ntRoot Using Basic Protocol 2, *^a^*

chrom	start	end	ancestry_prediction	AFR‐score	AMR‐score	EAS‐score	EUR‐score	SAS‐score
1	1	5000000	EUR	0.0174	0.0190	0.0173	0.0194	0.0188
1	5000001	10000000	AMR	0.0186	0.0191	0.0185	0.0190	0.0182
1	10000001	15000000	EUR	0.0154	0.0165	0.0153	0.0165	0.0162
1	15000001	20000000	EUR	0.0176	0.0185	0.0174	0.0185	0.0178
1	20000001	25000000	EUR	0.0172	0.0199	0.0184	0.0203	0.0194
1	25000001	30000000	EUR	0.0158	0.0176	0.0156	0.0176	0.0164
1	30000001	35000000	EUR	0.0174	0.0190	0.0174	0.0192	0.0187
1	35000001	40000000	EUR	0.0171	0.0189	0.0168	0.0196	0.0191
1	40000001	45000000	EAS	0.0166	0.0179	0.0182	0.0180	0.0178
1	45000001	50000000	SAS	0.0175	0.0184	0.0171	0.0185	0.0185

a Super‐population label abbreviations: AMR, Admixed American; AFR, African; EUR, European; SAS, South Asian; EAS, East Asian.

## Basic Protocol 3 RUNNING *ntRoot* WITH WHOLE EXOME SEQUENCING DATA

In Basic Protocol 3, we demonstrate using *ntRoot* to infer genetic ancestry from WES data. This protocol is similar to running *ntRoot* with WGS (Basic Protocol 1), except for setting a hard lower *k*‐mer multiplicity threshold and hard‐masking (convert bases to “N”) regions of the input reference genome. By default, ntStat will use *k*‐mer frequency profiles from ntCard to automatically determine an appropriate lower *k*‐mer multiplicity threshold from the input data. This reduces the impact of error *k*‐mers, which will occur less frequently than true genomic *k*‐mers. However, because the approach ntStat uses for this automatic threshold determination assumes that the data is WGS, it can set spuriously high *k*‐mer multiplicity cutoffs when analyzing WES. Therefore, when using WES input data, we set a lower *k*‐mer multiplicity threshold of 2. In addition, to ensure that the ntEdit SNV‐calling step focuses on the genic regions covered by the WES, we pre‐process the input reference genome to hard mask (convert bases to “N”) sequences that are outside of the genic regions targeted by the WES. As a result, ntEdit will only call SNVs over the regions covered by the exome sequencing, thus reducing spurious SNV calls. Then, *ntRoot* will proceed as in Basic Protocol 1, utilizing the detected SNVs as well as the IVC file from the 1000 Genomes Project to infer the genetic ancestry of the input sample.

### Necessary Resources

#### Hardware

64‐bit Linux or macOS operating system with sufficient RAM and diskspace (see Table 1)

##### Software

The listed software should be installed:

ntRoot (v1.2.4+): https://github.com/BirolLab/ntRoot
ntEdit (v2.1.1+): https://github.com/BirolLab/ntedit
bedtools: https://bedtools.readthedocs.io/en/latest/
snakemake: https://snakemake.readthedocs.io/en/stable/
samtools: https://www.htslib.org/
wget: https://www.gnu.org/software/wget/
gzip: https://www.gzip.org/
sed: https://www.gnu.org/software/sed/manual/sed.html
*For step‐by‐step instructions describing how to install these dependencies, please see the Support Protocol. Ensure that all dependencies are available in your PATH environment variable*.

##### Files

Whole exome sequencing reads (in FASTQ format)

Human reference genome (in FASTA format)

BED (Browser Extensible Data) file with the coordinates of the exome target regions on the human reference genome

IVC file from the 1000 Genomes Project (in VCF format)

*All files can be uncompressed or gzipped. The expected format for the IVC file is shown in* *Figure* *1*.

1 Prepare the working directory.


mkdir ntroot3 && cd ntroot3

2 Download the example whole exome sequencing reads.


nohup wget ftp://ftp.sra.ebi.ac.uk/vol1/fastq/ERR050/ERR050736/ERR050736_1.fastq.gz &> ERR050736_1.log &
nohup wget ftp://ftp.sra.ebi.ac.uk/vol1/fastq/ERR050/ERR050736/ERR050736_2.fastq.gz &> ERR050736_2.log &

  These commands will download the paired‐end Illumina WES short reads supplied by the 1000 Genomes Project for sample HG00864, which has self‐reported East Asian ancestry (Zheng‐Bradley et al., 2017). The use of “nohup” will allow the read files to be downloaded simultaneously. Each file will take ∼3 min to download. Ensure that both files have finished downloading before moving onto the next step.
3 Download the human reference genome and IVC files.
  a. If you have already completed Basic Protocol 1 or Basic Protocol 2, use the human reference genome and IVC files downloaded and extracted previously. Make a soft link to the downloaded folder in this working directory.

ln -s ../ntroot1/ntroot_supplementary_zenodo

  If the directory ntroot_supplementary_zenodo is in a different directory to the one listed in the example command, adjust the path accordingly.
   
  b. If you do not already have the human reference genome and IVC set files downloaded, follow Basic Protocol 1, steps 3 and 4.
4 Download the example BED file listing the coordinates of the genomic regions targeted in the whole exome sequencing.


wget https://ftp.1000genomes.ebi.ac.uk/vol1/ftp/data_collections/1000_genomes_project/working/20190125_coords_exon_target/output_1000G_Exome.v1.bed

  This BED file is provided by the 1000 Genomes Project based on their whole exome sequencing protocol.
5 Rename the downloaded BED file to ensure chromosome naming consistency between this file and the IVC file.


cat output_1000G_Exome.v1.bed |sed 's/chr//g' > output_1000G_Exome.v1.renamed.bed

  The BED file has chromosome names chr1, chr2, etc., but the IVC file has chromosome names 1, 2, etc. This command ensures consistent naming between the files.
6 Run *ntRoot* using the downloaded whole exome sequencing data.


ntroot --exome --reference ntroot_supplementary_zenodo/data/GRCh38.fa.gz --reads ERR050736 -t 24 --exome_bed output_1000G_Exome.v1.renamed.bed -l ntroot_supplementary_zenodo/data/1000GP_integrated_snv_v2a_27022019.GRCh38.phased_gt1.vcf.gz -k 55 --lai

  Before running the ntEdit SNV calling stage, ntRoot will use bedtools (Quinlan & Hall, 2010) to hard‐mask (convert to N's) the regions in the provided reference that are outside of the regions described in the input --exome_bed file. Then, this masked version of the reference file is used for the subsequent ntRoot steps. This step will take ∼10 min to run.
  When running future ntRoot commands that use whole exome sequencing reads with the same exome target regions, the masked reference genome can be supplied to ntRoot instead of using the BED file:


ntroot --exome --masked --reference masked_GRCh38.fa.gz --reads ERR050736 -l ntroot_supplementary_zenodo/data/1000GP_integrated_snv_v2a_27022019.GRCh38.phased_gt1.vcf.gz -k 55 -t 24 --lai

7 Check the *ntRoot* output log file to ensure that the process completed successfully.
  If the ntRoot run was successful, you should see the message “Done ntRoot!” at the end of your log file.
8 View the global ancestry inference file generated by *ntRoot*.
  The global ancestry inference results will be in the file ERR050736_ntedit_k55_exome_variants.vcf_ancestry-predictions_tile5000000.tsv. Detailed descriptions of the columns in this output file can be found in Table 2. The expected results are shown in Table 7. In this example, the global ancestry inference super‐population label is East Asian. There may be some minor differences in the numbers across different runs.

**Table 7:** Expected Global Ancestry Inference Results From Running ntRoot Using Whole Exome Sequencing Reads in Basic Protocol 3, *^a^*

GAI super‐population	LAI fraction (tile:5000000 bp)	GAI score	Total SNV count	Non‐zero AF SNV count
EAS	71.25%	0.5095	10878	10631
AFR	10.26%	0.4081	10878	10159
SAS	7.33%	0.4518	10878	10325
EUR	5.13%	0.4204	10878	10092
AMR	4.95%	0.4441	10878	10306
Unknown	1.10%	N/A	10878	N/A

a Super‐population label abbreviations: AMR, Admixed American; AFR, African; EUR, European; SAS, South Asian; EAS, East Asian.

9 View the local ancestry inference results generated by *ntRoot*.
  The local ancestry inference results from ntRoot can be found in the file ERR050736_ntedit_k55_exome_variants.vcf_ancestry-predictions-tile-resolution_tile5000000.tsv. For information about the columns in this output file, see Basic Protocol 1, step 8. The first 10 chromosome 1 tiles are shown in Table 8. There may be some minor differences in the numbers across different runs.

**Table 8:** The First 10 chromosome 1 Tiles in the Local Ancestry Inference Output File Generated by ntRoot Using Whole Exome Sequencing Reads in Basic Protocol 3, *^a^*

chrom	start	end	ancestry_prediction	AFR‐score	AMR‐score	EAS‐score	EUR‐score	SAS‐score
1	1	5000000	EAS	0.0175	0.0191	0.0224	0.0164	0.0198
1	5000001	10000000	EAS	0.0143	0.0142	0.0183	0.0130	0.0142
1	10000001	15000000	EAS	0.0096	0.0109	0.0135	0.0111	0.0117
1	15000001	20000000	EAS	0.0158	0.0167	0.0194	0.0165	0.0163
1	20000001	25000000	EAS	0.0163	0.0188	0.0204	0.0178	0.0185
1	25000001	30000000	EAS	0.0161	0.0175	0.0185	0.0150	0.0162
1	30000001	35000000	EAS	0.0123	0.0154	0.0156	0.0117	0.0137
1	35000001	40000000	EAS	0.0104	0.0121	0.0162	0.0112	0.0132
1	40000001	45000000	EAS	0.0138	0.0154	0.0179	0.0155	0.0164
1	45000001	50000000	EAS	0.0176	0.0199	0.0221	0.0191	0.0208

a Super‐population label abbreviations: AMR, Admixed American; AFR, African; EUR, European; SAS, South Asian; EAS, East Asian.

## Basic Protocol 4 RUNNING *ntRoot* WITH ILLUMINA SINGLE NUCLEOTIDE POLYMORPHISM ARRAY DATA

In Basic Protocol 4, we demonstrate how *ntRoot* can be used to infer genetic ancestry from SNP array data. SNP arrays are a cost‐effective and highly accurate approach for genotyping human samples and have found wide applications in cancer genomics (Song & Shao, 2016). Both Illumina and Affymetrix provide commercial SNP microarray kits which target ∼1 million SNPs in the human genome (LaFramboise, 2009). In this protocol, we demonstrate how to convert SNP array data from the Illumina Infinium Global Screening Array (GSA v4.0) to a standard VCF file. The VCF file is then input to *ntRoot* to infer genetic ancestry directly from the detected SNPs. This protocol can be applied to any other Illumina SNP array version, given that the required input files are available.

### Necessary Resources

#### Hardware

64‐bit Linux or macOS operating system with sufficient RAM and diskspace (see Table 1)

##### Software

The listed software should be installed:

ntRoot (v1.2.4+): https://github.com/BirolLab/ntRoot
bedtools: https://bedtools.readthedocs.io/en/latest/
bcftools: https://samtools.github.io/bcftools/bcftools.html
bcftools gtc2vcf plugin: https://github.com/freeseek/gtc2vcf
snakemake: https://snakemake.readthedocs.io/en/stable/
samtools: https://www.htslib.org/
wget: https://www.gnu.org/software/wget/
*For step‐by‐step instructions describing how to install these dependencies, please see the Support Protocol. Information about installing the bcftools gtc2vcf plugin is included in steps 2 and 3 of this protocol. Ensure that all dependencies are available in your PATH environment variable*.

##### Files

Illumina SNP array data from the Infinium Global Screening Array v4.0

Human reference genome (in FASTA format)

IVC file (in VCF format)

*The expected format for the IVC file is shown in* *Figure* *1* *. The Illumina SNP array data files are in IDAT, BMP, EGT and CSV (comma‐separated value) formats. Information about the Illumina SNP array‐specific input file formats (IDAT, BMP, EGT) can be found at*: https://www.illumina.com/content/dam/illumina‐marketing/documents/products/technotes/technote_array_analysis_workflows.pdf.

1 Prepare the working directory.


mkdir ntroot4 && cd ntroot4

2 Check that bcftools (Danecek et al., 2021) and the bcftools gtc2vcf plugins are installed properly.


bcftools --help
bcftools plugin -l

  After running the first command, a help page for bcftools should be printing to your console. The second command will list the available bcftools plugins; ensure that “idat2gtc” is listed. If it is, proceed to step 4. Otherwise, the instructions for installing and configuring the plugin are explained in step 3.
3 Install and configure the bcftools gtc2vcf plugin.
  a. Option 1: Using conda (recommended, Linux only).

conda install -c conda-forge -c bioconda bcftools-gtc2vcf-plugin

  After installing the plugin, deactivate and reactivate your conda environment. Then, run this command again:


bcftools plugin -l

  The “idat2gtc” plugin should now be listed.
   
  b. Option 2: Use pre‐compiled binary (Linux only).

wget https://software.broadinstitute.org/software/gtc2vcf/gtc2vcf_1.22-20250819.zip
unzip gtc2vcf_1.22-20250819.zip
export BCFTOOLS_PLUGINS=$PWD
bcftools plugin -l

  These commands will download the pre‐compiled binary, uncompress it, and adjust the environment flag to point to the downloaded binaries. Finally, check that the plugin “idat2gtc” appears in the list when running the bcftools plugin command.
   
  c. Option 3: Use a Docker image for the bcftools steps.

apptainer pull docker://quay.io/biocontainers/bcftools-gtc2vcf-plugin:1.22--hb66fcc3_0

  The above command is an example of fetching the docker image using Apptainer. Then, when running the bcftools steps, add the appropriate Apptainer exec options before the command. More information about the Apptainer command structure can be found in the Support Protocol. You will also need to set the BCFTOOLS_PLUGINS environment flag to point to the location of the gtc2vcf plugin. An example for Apptainer is shown below. The path may vary based on your operating system; if needed, use apptainer shell to enter an interactive terminal session to find the correct path.


export APPTAINER_BCFTOOLS_PLUGIN=/usr/local/libexec/bcftools

  If desired, Docker can also be used with the above bcftools Docker image.
   
  d. Option 4: Compile from source code.

wget http://github.com/samtools/bcftools/releases/download/1.22/bcftools-1.22.tar.bz2
tar xjvf bcftools-1.22.tar.bz2 && cd bcftools-1.22
wget -P plugins http://raw.githubusercontent.com/freeseek/gtc2vcf/master/{idat2gtc.c,gtc2vcf.{c,h},affy2vcf.c,BAFregress.c}
make
export BCFTOOLS_PLUGINS=$(pwd)/plugins && export PATH=$(pwd):$PATH && cd ../

  The above commands will download the source code for bcftools, then download and compile the gtc2vcf plugin.
4 Download the human reference genome and IVC files.
  a. If you have already completed Basic Protocol 1, 2 or 3, use the human reference genome and IVC files downloaded and extracted previously. Make a soft link to the downloaded folder in this working directory.

ln -s ../ntroot1/ntroot_supplementary_zenodo

  If the directory ntroot_supplementary_zenodo is in a different directory to the one listed in the example command, adjust the path accordingly.
   
  b. If you do not already have the human reference genome and IVC files downloaded, follow Basic Protocol 1, steps 3 and 4.
5 Make an uncompressed copy of the human reference genome for subsequent bcftools commands.


gunzip -c ntroot_supplementary_zenodo/data/GRCh38.fa.gz > GRCh38.fa

6 Download the example Illumina SNP array data.


wget https://support.illumina.com/content/dam/illumina-support/documents/documentation/chemistry_documentation/infinium_assays/infinium-gsa-with-gcra/idats_Demo_36.zip
wget https://support.illumina.com/content/dam/illumina-support/documents/documentation/chemistry_documentation/infinium_assays/infinium-gsa-with-gcra/GSA-48v4-0_20085471_D2.bpm
wget https://support.illumina.com/content/dam/illumina-support/documents/documentation/chemistry_documentation/infinium_assays/infinium-gsa-with-gcra/InfiniumGlobalScreeningArrayv4.0ClusterFile-egt.zip
wget https://support.illumina.com/content/dam/illumina-support/documents/documentation/chemistry_documentation/infinium_assays/infinium-gsa-with-gcra/GSA-48v4-0_20085471_D2.csv

7 Uncompress the zipped downloaded files.


unzip idats_Demo_36.zip
unzip InfiniumGlobalScreeningArrayv4.0ClusterFile-egt.zip

8 Convert the IDAT SNP array files to GTC format using bcftools.


bcftools +idat2gtc --bpm GSA-48v4-0_20085471_D2.bpm --egt GSA-48v4-0_20085471_D2_ClusterFile.egt --idats idats_Demo_36/206956030018/ --output ./samples_206956030018/

9 Convert the GTC file to BCF format using bcftools


bcftools +gtc2vcf --no-version -Ou --bpm GSA-48v4-0_20085471_D2.bpm --csv GSA-48v4-0_20085471_D2.csv --egt GSA-48v4-0_20085471_D2_ClusterFile.egt --gtcs samples_206956030018/ --fasta-ref GRCh38.fa --extra samples_206956030018_out.tsv --threads 24 | bcftools sort -Ou -Tidats_Demo_36/206956030018/bcftools.tmp | bcftools norm --no-version -Ob -o samples_206956030018.bcf -c x -f GRCh38.fa --write-index

10 Only retain variant calls for sample R16C03 and select variants with a non‐reference base (homozygous or heterozygous).


bcftools view -Oz -o R16C03.vcf -i 'ALT!="." && GT="alt"' --samples 206956030018_R16C03 samples_206956030018.bcf

11 Run *ntRoot* using the VCF file generated from the SNP array data.


ntroot -r GRCh38.fa --custom_vcf R16C03.vcf -l ntroot_supplementary_zenodo/data/1000GP_integrated_snv_v2a_27022019.GRCh38.phased_gt1.vcf.gz --lai -t 24

  This command will take ∼7 min to complete.
12 Check the *ntRoot* output log file to ensure that the process completed successfully.
  If the ntRoot run was successful, you should see the message “Done ntRoot!” at the end of your log file.
13 View the global ancestry inference file generated by *ntRoot*.
  The global ancestry inference results can be found in the file R16C03.vcf.cross-ref.vcf_ancestry-predictions_tile5000000.tsv. The expected results are shown in Table 9, and information about the output columns are described in Table 2. In this example, the predicted GAI ancestry label is European.

**Table 9:** Expected Global Ancestry Inference Results From Running ntRoot Using Illumina SNP Array Data in Basic Protocol 4, *^a^*

GAI super‐population	LAI fraction (tile:5000000 bp)	GAI score	Total SNV count	Non‐zero AF SNV count
EUR	74.60%	0.4641	152710	152675
AMR	11.46%	0.4523	152710	151696
AFR	5.82%	0.4077	152710	145138
SAS	4.76%	0.4444	152710	149458
EAS	3.35%	0.4220	152710	144404

a Super‐population label abbreviations: AMR, Admixed American; AFR, African; EUR, European; SAS, South Asian; EAS, East Asian.

14 View the local ancestry inference results generated by *ntRoot*.
  The local ancestry inference results from ntRoot can be found in the file R16C03.vcf.cross-ref.vcf_ancestry-predictions-tile-resolution_tile5000000.tsv. For information about the columns in this output file, see Basic Protocol 1, step 8. The first 10 chromosome 1 tiles from this file are shown in Table 10.

**Table 10:** The First 10 Chromosome 1 Tiles From the Local Ancestry Inference Results Generated by ntRoot Using Illumina SNP Array Data in Basic Protocol 4, *^a^*

chrom	start	end	ancestry_prediction	AFR‐score	AMR‐score	EAS‐score	EUR‐score	SAS‐score
1	1	5000000	EUR	0.0167	0.0176	0.0169	0.0181	0.0180
1	5000001	10000000	EUR	0.0181	0.0181	0.0180	0.0184	0.0183
1	10000001	15000000	EUR	0.0161	0.0176	0.0161	0.0181	0.0170
1	15000001	20000000	EUR	0.0164	0.0178	0.0173	0.0185	0.0172
1	20000001	25000000	EUR	0.0159	0.0184	0.0181	0.0190	0.0186
1	25000001	30000000	EUR	0.0159	0.0187	0.0185	0.0189	0.0180
1	30000001	35000000	EUR	0.0164	0.0170	0.0163	0.0178	0.0175
1	35000001	40000000	EUR	0.0173	0.0194	0.0187	0.0200	0.0190
1	40000001	45000000	EUR	0.0152	0.0174	0.0159	0.0181	0.0168
1	45000001	50000000	EUR	0.0145	0.0184	0.0153	0.0203	0.0174

a Super‐population label abbreviations: AMR, Admixed American; AFR, African; EUR, European; SAS, South Asian; EAS, East Asian.

## Basic Protocol 5 RUNNING *ntRoot* WITH VARIANT CALL FORMAT FILES FROM VARIANT CALLING PIPELINES

Although the *ntRoot* pipeline can natively perform variant calling, it can also infer genetic ancestry using SNVs identified by a different variant calling pipeline. Many genetic workflows will perform variant calling for other analyses, so the ability to infer genetic ancestry directly from this pre‐existing file is value added. In Basic Protocol 5, we demonstrate how to use *ntRoot* to perform this analysis. Given a standard VCF file from any variant calling pipeline, *ntRoot* will cross‐reference the SNVs with the IVC file, then infer the genetic ancestry.

### Necessary Resources

#### Hardware

64‐bit Linux or macOS operating system with sufficient RAM and diskspace (see Table 1)

##### Software

The listed software should be installed:

ntRoot (v1.2.4+): https://github.com/BirolLab/ntRoot
bedtools: https://bedtools.readthedocs.io/en/latest/
snakemake: https://snakemake.readthedocs.io/en/stable/
samtools: https://www.htslib.org/
wget: https://www.gnu.org/software/wget/
*For step‐by‐step instructions describing how to install these dependencies, please see the Support Protocol. Ensure that all dependencies are available in your PATH environment variable*.

##### Files

VCF file from a variant calling pipeline

Matching human reference genome (in FASTA format)

IVC file from the 1000 Genomes Project (in VCF format)

*All files can be uncompressed or gzipped. The expected format for the IVC file is shown in* *Figure* *1*.

1 Prepare the working directory.


mkdir ntroot5 && cd ntroot5

2 Download the example VCF file.


wget https://zenodo.org/records/22235499/files/ERR3242132.all-chr.renamed.vcf.gz

  To illustrate running ntRoot using a VCF file from a third‐party variant calling pipeline, we use a VCF file produced using WGS reads from 1000 Genomes Project sample HG00864, which has self‐reported East Asian ancestry (Zheng‐Bradley et al., 2017). This VCF file was generated by aligning WGS reads to the human reference using bwa mem (Li, 2013), then calling variants using GATK HaplotypeCaller (Van der Auwera & O'Connor, 2020).
3 Download the human reference genome and IVC file.
  a. If you have already completed any of Basic Protocols 1 to 4, use the human reference genome and IVC files downloaded and extracted previously. Make a soft link to the downloaded folder in this working directory.

ln -s ../ntroot1/ntroot_supplementary_zenodo

  If the directory ntroot_supplementary_zenodo is in a different directory to the one listed in the example command, adjust the path accordingly.
   
  b. If you do not already have the human reference genome and IVC files downloaded, follow Basic Protocol 1, steps 3 and 4.
4 Run *ntRoot*.


ntroot --custom_vcf ERR3242132.all-chr.renamed.vcf.gz --reference ntroot_supplementary_zenodo/data/GRCh38.fa.gz -l ntroot_supplementary_zenodo/data/1000GP_integrated_snv_v2a_27022019.GRCh38.phased_gt1.vcf.gz --lai -t 24

  The ntRoot command will take ∼10 min to complete.
5 Check the *ntRoot* output log file to ensure that the process completed successfully.
  If the ntRoot run was successful, you should see the message “Done ntRoot!” at the end of your log file.
6 View the global ancestry inference file generated by *ntRoot*.
  The global ancestry inference results can be found in the file ERR3242132.all-chr.renamed.vcf.gz.cross-ref.vcf_ancestry-predictions_tile5000000.tsv. The expected results are shown in Table 11, and information about the columns of this file are described in Table 2. In this example, the global genetic ancestry super‐population label would be East Asian.

**Table 11:** Expected Results From Running ntRoot in Basic Protocol 5 Using the VCF File Generated Using a Third‐Party Variant Calling Pipeline*^a^*

GAI super‐population	LAI fraction (tile:5000000 bp)	GAI score	Total SNV count	Non‐zero AF SNV count
EAS	97.19%	0.5409	3726126	3717741
SAS	1.05%	0.4859	3726126	3648437
AFR	0.88%	0.4379	3726126	3580151
AMR	0.70%	0.4796	3726126	3639526
EUR	0.18%	0.4596	3726126	3591175

a Super‐population label abbreviations: AMR, Admixed American; AFR, African; EUR, European; SAS, South Asian; EAS, East Asian.

7 View the local ancestry inference file generated by *ntRoot*.
  The local ancestry inference results generated by ntRoot can be found in the file ERR3242132.all-chr.renamed.vcf.gz.cross-ref.vcf_ancestry-predictions-tile-resolution_tile5000000.tsv. For information about the columns in this output file, see Basic Protocol 1, step 8. The first 10 lines of the expected results from this file can be seen in Table 12.

**Table 12:** The First 10 Chromosome 1 Tiles in the Local Ancestry Inference Results Generated by Running ntRoot in Basic Protocol 5 Using the VCF File Generated Using a Third‐Party Variant Calling Pipeline*^a^*

chrom	start	end	ancestry_prediction	AFR‐score	AMR‐score	EAS‐score	EUR‐score	SAS‐score
1	1	5000000	EAS	0.0171	0.0185	0.0209	0.0173	0.0189
1	5000001	10000000	EAS	0.0192	0.0195	0.0226	0.0188	0.0193
1	10000001	15000000	EAS	0.0149	0.0158	0.0184	0.0152	0.0163
1	15000001	20000000	EAS	0.0172	0.0190	0.0217	0.0185	0.0186
1	20000001	25000000	EAS	0.0175	0.0206	0.0231	0.0203	0.0208
1	25000001	30000000	EAS	0.0176	0.0197	0.0227	0.0188	0.0204
1	30000001	35000000	EAS	0.0160	0.0170	0.0196	0.0162	0.0180
1	35000001	40000000	EAS	0.0151	0.0168	0.0195	0.0150	0.0164
1	40000001	45000000	EAS	0.0160	0.0173	0.0203	0.0171	0.0180
1	45000001	50000000	EAS	0.0177	0.0197	0.0226	0.0191	0.0203

a Super‐population label abbreviations: AMR, Admixed American; AFR, African; EUR, European; SAS, South Asian; EAS, East Asian.

## INSTALLING DEPENDENCIES FOR THE BASIC PROTOCOLS

In this Support Protocol, we will provide step‐by‐step instructions for installing all dependencies required for running the *ntRoot* pipelines described in the Basic Protocols. We recommend installing the dependencies using the conda package manager (Option A). Alternatively, *ntRoot* can be run using the Docker image provided on Quay.io (Option B).

### Necessary Resources

#### Hardware

64‐bit Linux or macOS operating system with sufficient diskspace

##### Software

Directions for installing all required software are outlined in the protocol steps

##### Files

None

#### Option A: Installing dependencies using conda (recommended)

1a If conda is not already installed, install it using miniforge. Otherwise, continue to step 2a.


wget https://github.com/conda-forge/miniforge/releases/latest/download/Miniforge3-$(uname)-$(uname -m).sh
bash Miniforge3-$(uname)-$(uname -m).sh

  These commands will download the installer, then run the installation. The interactive installation will configure and initialize conda. The above commands should download the correct installer for your operating system, but if there are any issues, download the appropriate installer at: https://github.com/conda‐forge/miniforge.
2a Create a new conda environment and activate the new environment.


conda create -n ntroot
conda activate ntroot

3a Install *ntRoot* and other required dependencies in the new conda environment.


conda install -c conda-forge -c bioconda ntroot wget sed gzip bcftools

  All other dependencies listed in the various protocols will be installed along with ntRoot with the conda command in step 3a. Alternatively, to install the versions of each dependency used in testing this protocol, use the conda environment file provided for this protocol.


wget https://zenodo.org/records/22235499/files/ntroot_protocol.conda-environment.yaml
conda install --file ntroot_protocol.conda-environment.yaml

#### Option B: Running ntRoot using the quay.io Docker image

1b Install Docker or Apptainer (formerly Singularity).
  Follow the instructions for installing Apptainer (https://apptainer.org/docs/admin/main/installation.html) or Docker (https://docs.docker.com/engine/install/). The following steps will use Apptainer.
2b Download the *ntRoot* Docker image.


apptainer pull docker://quay.io/biocontainers/ntroot:1.2.4--pl5321hdfd78af_1

  To download a different version of ntRoot, see: https://quay.io/repository/biocontainers/ntroot?tab=tags. All other dependencies required for the Basic Protocols are also available in this Docker image, except for the bcftools plugin required for Basic Protocol 4. See Basic Protocol 4, steps 2 and 3, for the bcftools plugin installation instructions.
3b When running any of the *ntRoot* commands, prepend the command with:


apptainer exec -B $PWD:$PWD ntroot_1.2.4--pl5321hdfd78af_1.sif <ntRoot command>

  Ensure that all directory paths needed by ntRoot, including the current working directory, directory containing input reference genome file, etc. are listed in a comma‐separated list after ‐B. The formatting for the current working directory is shown in the example command.

## COMMENTARY

### Background Information

Large‐scale genomic population studies employ a range of different data types, depending on the study's scope, budget and aims. Because genetic ancestry is such an important variable in these studies, being able to leverage the same data for ancestry inference is value‐added. As shown in this protocol, *ntRoot* is a scalable, streamlined tool that can be used to infer ancestry directly from whole genome sequencing (WGS), whole exome sequencing (WES), genome assemblies, SNP arrays, and variant files from standard variant calling pipelines. The output files are easy to parse and understand, making *ntRoot* a user‐friendly and accessible ancestry inference tool. While single samples were processed in the protocol steps to demonstrate the different modes in the *ntRoot* toolkit, the fast execution time and highly parallelizable per‐sample inference approach of *ntRoot* makes it well‐suited to analyzing any number of input samples. This scalability has been demonstrated in practice through the application of *ntRoot* to whole‐genome sequencing data from 10,250 participants in the Canadian HostSeq cohort (Warren et al., 2026).

In addition to supporting multiple input data types, *ntRoot* is also flexible to the integrated variant call set file used. While in this protocol we demonstrate the use of *ntRoot* with an IVC file generated by the 1000 Genomes Project with 5 super‐populations, *ntRoot* can easily be run using different variant sets, different reference populations, or even other species by changing the IVC file (parameter “‐l”) and, if applicable, the input reference genome. The supplied file just needs to adhere to the expected format, which is shown in Figure 1.

*ntRoot*’s scalability stems from its use of Bloom filters (Bloom, 1970) to detect SNVs, rather than relying on read alignments, which are used by many standard variant‐calling pipelines and commonly used ancestry‐inference workflows. Bloom filters are probabilistic data structures implemented as a bit array (0s and 1s), which support efficient set membership queries. Elements are added to the Bloom filter by first hashing them (using a hash function to convert the sequence to an integer) to determine which locations in the array to set to 1, indicating presence. Queries are performed by hashing the element and checking the corresponding array positions; 0 indicates the element is not present in the set, while a 1 indicates that the element is probably in the set. This probabilistic behavior is due to the possibility of hash collisions, where different elements generate the same hash value. In *ntRoot*, we leverage ntEdit to populate the Bloom filters with *k*‐mers from the sequencing reads or genome assemblies and then perform lookups to identify differences between the input data and the reference genome. Due to their underlying bit array structure and thus fast lookups, ntEdit is a fast and memory‐efficient approach for detecting SNVs, which are then cross‐referenced with the integrated variant call set to infer ancestry.

Furthermore, while other ancestry inference tools require many steps of data preparation prior to their use, *ntRoot* provides a streamlined workflow for variant detection directly from several commonly generated genomic data types. ADMIXTURE (Alexander et al., 2009) and STRUCTURE (Pritchard et al., 2000), two commonly‐used tools for ancestry inference, require genotype data as input and typically involve multiple upstream steps to generate and format those data. SNVstory (Bollas et al., 2024), a more recent ancestry inference tool, also requires variant calling to be performed prior to running their ancestry prediction pipeline. In contrast, *ntRoot* can perform alignment‐free variant detection directly from sequencing reads or genome assemblies before ancestry inference, while also supporting SNP array data and pre‐existing VCF files. In our tests with WGS, performing the short sequencing reads alignments required for state‐of‐the‐art ancestry inference tools alone took over 5× longer than the entire *ntRoot* pipeline using the same sequencing data (see Supporting Information, Supplementary Table 1).

With support for multiple input data types and a streamlined workflow, *ntRoot* is a fast, easy‐to‐use tool for small‐ or large‐scale ancestry inference, with fewer data pre‐processing requirements and shorter execution times than the ancestry‐inference workflows previously benchmarked (Warren et al., 2025), making it an accessible and versatile tool that can benefit a broad range of researchers across scientific communities.

### Critical Parameters

#### **‐k**

When using sequencing reads or a genome assembly with *ntRoot*, the variant calling step using ntEdit is a critical part of the pipeline. ntEdit uses a *k‐*mer guided approach for detecting SNVs, so the choice of *k* can have a large impact on the results. Generally, we find that setting ‐k 55 works well across the different input data types. However, in some cases, this value may need to be adjusted. For example, if the input sequencing reads are very short (<90 bp), the results may benefit from a lower “‐k” setting.

#### **‐t**

The number of threads specified using the “‐t” parameter, strongly influences the runtime of the *ntRoot* pipeline. For faster runtimes, we recommend setting the number of threads as high as feasible given the specifications and current workload of the machine.

#### --cutoff

The set of *k*‐mers from the input sequencing reads considered for SNV detection greatly influences the accuracy of variant detection with ntEdit. Generally, setting a lower threshold for *k*‐mer multiplicity (--cutoff), i.e., retaining only *k*‐mers found in the input reads at least --cutoff times, will filter out erroneous *k*‐mers. By default (--cutoff 0), ntCard and ntStat analyze the distribution of the input reads and determine a suitable threshold. However, --cutoff can also be used to set a hard threshold for *k*‐mer multiplicity. This is particularly important when analyzing WES, as shown in Basic Protocol 3, and when analyzing low‐coverage WGS data.

#### --strip_info

A VCF file output from *ntRoot* can be reanalyzed with a different integrated variant call set or different parameters using the --custom_vcf option. In this case, it is important to include the --strip_info parameter in the *ntRoot* command to avoid conflicting ancestral allele frequency annotations between the existing file and the new cross‐referenced alleles.

### Advanced Parameters

#### --tile

The --tile parameter controls the size (in bp) of genomic tiles used for the local ancestry inference, which in turn informs the global ancestry label. We have found the default setting (5 Mbp) to perform well in most cases, but users may adjust it depending on their input data and research workflow.

#### --recomb_bed

Optionally, a user can provide a BED file containing genomic tile coordinates, and these pre‐defined tiles will be used for global and local ancestry inference instead of fixed length tiles. This option gives advanced users the option of using variable‐length, recombination‐informed tiles for ancestry inference, for example.

#### **‐z**

By default, all sequences >100 bp are considered for ancestry inference. In practice, input chromosome sequences are typically much longer than this threshold, but users can control this filtering with the “‐z” parameter. In most cases, this parameter will not need to be adjusted; however, it can be set to exclude smaller chromosomes from the analysis, for example.

#### **‐j**

When running ntEdit to identify SNVs in the input data, the “‐j” parameter controls the spacing of the *k*‐mer subset used to query the presence or absence of base variants. By default, this parameter is set to 3, meaning every third *k*‐mer is inspected. We find that this default value preserves sensitivity while reducing runtime by decreasing the number of Bloom filter queries, but it can be adjusted if desired.

#### ‐X and ‐Y

The “‐X” and “‐Y” parameters control the fraction of *k*‐mers evaluated for a putative variant that are expected to be absent or present, respectively, for the variant to be reported. By default, these values are set to 0.5 and increasing them results in more stringent variant calling.

### Troubleshooting

For all *ntRoot* runs, it is important to carefully review the log file to ensure that the pipeline was successful. If there are any errors in the log file, these issues must be addressed before the results can be reliably analyzed. See Table 13 for common errors and solutions.

**Table 13:** Sources and Solutions to Potential Errors

Problem	Possible Cause	Solution
Error: ‐bash: ntroot: command not found	The *ntRoot* driver script was not found in your PATH environment variable	Find the location of your *ntRoot* installation, and prepend this path to your PATH environment variable: export PATH=/path/to/ntroot:$PATH
Error with messages such as “cannot run run‐ntedit: No such file or directory” and “Error in rule ntedit_reads:”	ntEdit cannot be found in your PATH environment variable	Add the location of your ntEdit installation to your PATH environment variable: export PATH=/path/to/ntedit/:$PATH
ArgumentTypeError after launching *ntRoot*	Using an incompatible combination of input parameters	Ensure that the specified parameters are appropriate for your *ntRoot* run. For example, the parameters --reads, --genome, and --custom_vcf should never be used together, as they indicate running *ntRoot* using sequencing reads, a genome assembly, and a third‐party VCF file, respectively; the error message will indicate which specified parameters are incompatible
The log file lists various commands, but no output files are seen in the working directory	Running an *ntRoot* “dry run”, where all the commands that would be run are printed, but they are not executed.	To execute all the commands and generate the output files, remove ‐n from the *ntRoot* command.
Messages in the log file like: “WARNING: chr11 tile25 has 80 only total SNVs – you may need to increase the tile size (currently set at 5000000)”	Some regions of the genome have fewer than expected ancestry‐determinant SNVs detected	When running *ntRoot* using WES, messages like this are expected, since only SNVs in exonic regions will be detected; generally, if there are enough SNVs identified (see Understanding Results), no further action is needed
When using --custom_vcf, the output global ancestry file only contains the header line	Chromosome naming inconsistencies between the input VCF file and the IVC file	Compare the chromosome naming between the VCF files, and ensure they are consistent, renaming one of the files if needed; a common reason for this inconsistency is when the input VCF file uses the format *chr1, chr2*, etc., but the IVC file uses the format *1, 2*, etc.
Error “No such file or directory” in log file when running *ntRoot* commands using Apptainer	Some input files are in directories which are not specified properly in the apptainer command using the ‐B parameter	Include the directory paths for all input files, including the target locations for soft‐linked files, in the ‐B parameter for the apptainer command; see the Support Protocol (Option B) for an example
When specifying --exome_bed, an error in the log file which points to “localrule exome_sort_bed”, with an error message in the form “Chromosome "chr1" undefined in GRCh38.fa.gz.fai”	Naming inconsistencies between the reference genome FASTA file and the --exome_bed BED file	Rename the chromosomes in the BED file to match the reference human genome FASTA file; this error is commonly caused by the BED file using the chromosome naming convention *chr1, chr2*, etc., while the reference FASTA genome uses *1, 2*, etc.

### Understanding Results

For each of the protocols, *ntRoot* will output files which summarize both the global and local ancestry inferences from the input data. If the final files with the ancestry fraction predictions (*_ancestry‐predictions_tile5000000.tsv and *_ancestry‐predictions‐tile‐resolution_tile5000000.tsv) are generated in the working directory, this indicates that the run completed successfully. The predicted super‐population labels are defined by the provided IVC file. The IVC file used in this protocol includes 5 continental labels: African (AFR), Admixed American (AMR), European (EUR), South Asian (SAS), and East Asian (EAS). Any genomic tiles with ambiguous ancestry will be assigned the label “Unknown”. *ntRoot* will also output an interactive HTML file (*ntroot‐lai‐interactive_tile5000000.html) that summarizes the global and local ancestry results.

The global ancestry inference summary file provides estimated genome‐wide ancestry fractions for each ancestral population, as well as a “GAI score”, as detailed in Table 2. Because the ancestral fractions are calculated from the proportions of pre‐determined genomic tiles assigned to each ancestry, the reported fractions may deviate from underlying ancestry proportions and should not necessarily be interpreted as calibrated admixture proportions. Generally, if a user wishes to assign a single genetic ancestry label to the input sample, we recommend using the population with the highest LAI‐derived ancestry fraction, which corresponds to the populations ordered by decreasing fraction in the file. However, if multiple ancestries have very similar ancestry fractions (within ∼5%), or there is a substantial “Unknown” fraction (>20%), it may be more appropriate to assign multiple genetic ancestry labels to that sample. This can provide a more descriptive label for individuals with admixed ancestry. These thresholds are intended as practical guidance rather than validated statistical cutoffs.

The local ancestry inference summary provides super‐population assignments for each 5 Mbp (by default) tile of the genome. This allows for a more nuanced analysis of the underlying ancestral population across genomic segments, while the global ancestry (i.e., GAI) assigns an overall label for the input sample. Unlike haplotype‐based local ancestry methods which utilize phased genomic data and assign ancestry to variable‐length genome segments, *ntRoot* utilizes unphased data for local ancestry inference and, by default, uses fixed‐length tiles (Avadhanam & Williams, 2025). Optionally, users can supply a BED file to define variable‐sized genome tiles for the ancestry analysis (see Advanced Parameters).

The global ancestry inference file also tallies the number of SNVs used for the ancestry inference, which is useful for assessing the confidence of the ancestry inferences. Because *ntRoot* relies on the detected SNVs for inferring ancestry, if the number of SNVs detected is very low (<10,000), this can lead to less confident ancestry inferences. This is most likely to occur when using exome sequencing, since these sequencing reads target a narrower genetic landscape (<2%) as compared to whole genome sequencing. In particular, while the predicted global ancestry super‐population label remains robust to a low number of SNVs, the fractions of the various super‐populations may be less reliable. The confidence of the ancestry inference can also be impacted by low coverage WGS (<5‐fold); when using *ntRoot* with this data, we recommend setting --cutoff 1 to ensure that a sufficient set of *k*‐mers are retained for SNV identification. In our experiments, we have found that the ancestry inferences become less accurate when using <1× WGS coverage.

*ntRoot* will also output various intermediate files, depending on what mode is being run. When running *ntRoot* with WGS, WES, or genome assemblies (Basic Protocols 1 to 3), the SNVs detected by ntEdit will be output in VCF format, in addition to the final ancestry prediction files. This file can be useful in analyzing which SNVs detected by ntEdit are found in the reference IVC file and thus inform genetic ancestry inference. This information is captured in the INFO column, where the allele frequencies for the various super‐populations are indicated using tags in the format “<super population>_AF:<allele frequency>”. When using VCF files from SNP arrays or a third‐party variant calling pipeline, *ntRoot* also outputs an intermediate VCF file with this cross‐referenced information in the same format.

### Author Contributions

**Lauren Coombe**: Conceptualization; data curation; formal analysis; investigation; methodology; software; validation; writing—original draft; writing—review and editing. **Taghrid Aloraini**: Validation; writing—review and editing. **Inanc Birol**: Funding acquisition; supervision; writing—review and editing. **René Warren**: Conceptualization; investigation; methodology; software; supervision; writing—review and editing.

### Conflict of Interest

The authors declare that they have no conflicts of interest.

## Supporting information




*Supplementary Table 1 contains benchmarking results from running ntRoot and SNVstory ancestry inference pipelines using WGS data from sample HG00864. Supplementary Table 2 contains Accessions and URLs for the sample data used in the protocols*.

## Data Availability

The accessions and URLs for the sample data used in this protocol are specified throughout the protocol steps and can also be found in Supplementary Table 2 (see Supporting Information).
