# Supplementary material for: ntRoot: Genetic Ancestry Inference Across Diverse Genomic Data Types
Source: Curr Protoc. 2026 Oct 29;6(11):e70491. doi: 10.1002/cpz1.70491 (PMC13717937; doi:10.1002/cpz1.70491)
Supplement: Supplementary file 1 [file CPZ1-6-0-s001.pdf]

Supporting Information for "ntRoot: Genetic ancestry inference across diverse genomic data types"

Lauren Coombe, Taghrid Aloraini, Inanc Birol, René L. Warren

Supplementary Table 1: Benchmarking results from running ntRoot and SNVstory ancestry inference pipelines using WGS data from sample HG00864.

Ancestry inference pipeline	Threads	Step	Wall-clock time (min)
**ntRoot (Warren et al., 2025)**	24	Full pipeline (variant calling + ancestry inference)	69.55
**SNVstory (Bollas et al., 2024)**	24	Alignment of reads to the human genome reference using BWA mem (Li, 2013)	351.76
		Variant calling using GATK HaplotypeCaller (Van der Auwera and O'Connor, 2020)[1]	111.81
		Ancestry inference	6.10

[1]Variant calling was run for each chromosome in parallel. The runtime for the longest-running job is shown.

Supplementary Table 2: Accessions and URLs for sample data used in the protocols.

Data	Basic Protocol	Accession/URL
GRCh38 human reference genome	1-5	https://zenodo.org/records/10976332/files/ntroot_supplementary_zenodo.tar.gz
Integrate variant call set	1-5	https://zenodo.org/records/10976332/files/ntroot_supplementary_zenodo.tar.gz
Whole genome shotgun sequencing reads from sample HG00864	1	ERR3242132
HuRef genome assembly	2	GCA_000002125.1
Whole exome sequencing reads from sample HG00864	3	ERR050736
1000 Genomes exome target BED file	3	https://ftp.1000genomes.ebi.ac.uk/vol1/ftp/data_collections/1000_genomes_project/working/20190125_coords_exon_target/output_1000G_Exome.v1.bed
Illumina SNP array IDAT files	4	https://support.illumina.com/content/dam/illumina-support/documents/documentation/chemistry_documentation/infinium_assays/infinium-gsa-with-gcra/idats_Demo_36.zip
Illumina SNP array BPM files	4	https://support.illumina.com/content/dam/illumina-support/documents/documentation/chemistry_documentation/infinium_assays/infinium-gsa-with-gcra/GSA-48v4-0_20085471_D2.bpm
Illumina SNP array EGT files	4	https://support.illumina.com/content/dam/illumina-support/documents/documentation/chemistry_documentation/infinium_assays/infinium-gsa-with-gcra/InfiniumGlobalScreeningArrayv4.0ClusterFile-egt.zip
Illumina SNP array CSV files	4	https://support.illumina.com/content/dam/illumina-support/documents/documentation/chemistry_documentation/infinium_assays/infinium-gsa-with-gcra/GSA-48v4-0_20085471_D2.csv
VCF file for sample HG00864	5	https://zenodo.org/records/19474195/files/ERR3242132.all-chr.renamed.vcf.gz

References

Bollas, A. E., Rajkovic, A., Ceyhan, D., Gaither, J. B., Mardis, E. R., and White, P. 2024. SNVstory: inferring genetic ancestry from genome sequencing data. *BMC bioinformatics* 25:76.

Li, H. 2013. Aligning sequence reads, clone sequences and assembly contigs with BWA-MEM. Available at: https://arxiv.org/abs/1303.3997.

Van der Auwera, G. A., and O'Connor, B. D. 2020. Genomics in the cloud: using Docker, GATK, and WDL in Terra. O'Reilly Media.

Warren, R. L., Coombe, L., Wong, J., Kazemi, P., and Birol, I. 2025. ntRoot: computational inference of human ancestry at scale from genomic data. *Bioinformatics Advances* 5:vbaf287.
